# Genomic investigations of successful invasions: the picture emerging from recent studies

**DOI:** 10.1111/brv.70005

**Published:** 2025-02-16

**Authors:** Joanna Kołodziejczyk, Anna Fijarczyk, Ilga Porth, Piotr Robakowski, Noel Vella, Adriana Vella, Agnieszka Kloch, Aleksandra Biedrzycka

**Affiliations:** ^1^ Institute of Nature Conservation, Polish Academy of Sciences Mickiewicza 33 Kraków 31‐120 Poland; ^2^ Natural Resources Canada, Laurentian Forestry Centre 1055 Rue du Peps Québec City Quebec G1V 4C7 Canada; ^3^ Department of Biology Laval University 1045 Avenue de la Médecine Québec City Quebec G1V 0A6 Canada; ^4^ Institute of Integrative Biology and Systems Laval University 1030 Avenue de La Médecine Québec City Quebec G1V 0A6 Canada; ^5^ Department of Wood and Forest Sciences Laval University 1030 Avenue de La Médecine Québec City Quebec G1V 0A6 Canada; ^6^ Centre for Forest Research Laval University 2405 Rue de La Terrasse Québec City Quebec G1V 0A6 Canada; ^7^ Faculty of Forestry and Wood Technology Poznań University of Life Sciences 71E Wojska Polskiego Street Poznań PL 60‐625 Poland; ^8^ Conservation Biology Research Group, Department of Biology University of Malta Msida MSD2080 Malta; ^9^ Faculty of Biology University of Warsaw Miecznikowa 1 Warsaw 02‐089 Poland

**Keywords:** biological invasions, invasive species, adaptation, admixture, selection, next‐generation sequencing, genomic diversity

## Abstract

Invasion biology aims to identify traits and mechanisms that contribute to successful invasions, while also providing general insights into the mechanisms underlying population expansion and adaptation to rapid climate and habitat changes. Certain phenotypic attributes have been linked to successful invasions, and the role of genetics has been critical in understanding adaptation of invasive species. Nevertheless, a comprehensive summary evaluating the most common evolutionary mechanisms associated with successful invasions across species and environments is still lacking. Here we present a systematic review of studies since 2015 that have applied genomic tools to investigate mechanisms of successful invasions across different organisms. We examine demographic patterns such as changes in genomic diversity at the population level, the presence of genetic bottlenecks and gene flow in the invasive range. We review mechanisms of adaptation such as selection from standing genetic variation and *de novo* mutations, hybridisation and introgression, all of which can have an impact on invasion success. This comprehensive review of recent articles on the genomic diversity of invasive species led to the creation of a searchable database to provide researchers with an accessible resource. Analysis of this database allowed quantitative assessment of demographic and adaptive mechanisms acting in invasive species. A predominant role of admixture in increasing levels of genetic diversity enabling molecular adaptation in novel habitats is the most important finding of our study. The “genetic paradox” of invasive species was not validated in genomic data across species and ecosystems. Even though the presence of genetic drift and bottlenecks is commonly reported upon invasion, a large reduction in genomic diversity is rarely observed. Any decrease in genetic diversity is often relatively mild and almost always restored *via* gene flow between different invasive populations. The fact that loci under selection are frequently detected suggests that adaptation to novel habitats on a molecular level is not hindered. The above findings are confirmed herein for the first time in a semi‐quantitative manner by molecular data. We also point to gaps and potential improvements in the design of studies of mechanisms driving rapid molecular adaptation in invasive populations. These include the scarcity of comprehensive studies that include sampling from multiple native and invasive populations, identification of invasion sources, longitudinal population sampling, and the integration of fitness measures into genomic analyses. We also note that the potential of whole genome studies is often not exploited fully in predicting invasive potential. Comparative genomic studies identifying genome features promoting invasions are underrepresented despite their potential for use as a tool in invasive species control.

## I. INTRODUCTION

Present‐day ecosystems are exposed to many threats from organisms that establish populations outside their natural range. While the transportation of live organisms around the world has increased dramatically since the beginning of the Industrial Revolution and is still on the rise (Bellard, Cassey & Blackburn, 2016; Zenetos & Galanidi, 2020), only some alien species become invasive (Mack *et al*., 2000; Seebens *et al*., 2017; Williamson & Fitter, 1996). According to the IUCN definition, invasive alien species are species that cause severe ecological or economic damage, with documented ecosystem impacts ranging from habitat destruction and disease transmission to displacement or even extinction of native species (Dueñas *et al*., 2021; Fortič *et al*., 2023; Molnar *et al*., 2008). Therefore, a major focus in invasion biology is to identify mechanisms that contribute to invasion success (Fournier *et al*., 2019; Parker *et al*., 2013). Predicting which species are likely to become invaders before their introduction outside their native range has been a prime objective of invasion biology. The comparison of phenotypic features of native and invasive populations has enabled the identification of intraspecific differences in phenotypes that facilitate invasion success (Hiatt & Flory, 2020). Native and invasive phenotypes arise from the interaction between genotype and environment in their respective populations and the impact of stochastic events. Therefore, examining phenotypic, genetic, and ecological differences across native and invaded environments is essential for understanding the mechanisms driving successful invasions.

In addition to the practical value of genetic studies for informing invasive species management, research uncovering invasive traits offers broader insights into the mechanisms of population expansion and adaptation to rapid climate and habitat change (Moran & Alexander, 2014). The most frequently cited hypotheses explaining invasion success highlight the roles of transport opportunity, propagule pressure, habitat matching, fecundity and population size as prerequisites and accelerators of species invasions. Invasive species often have significantly higher values for performance‐related traits, better dispersal abilities, and shorter generation times compared to native species (Jeschke, 2014).

Species with low adaptive potential, that is a limited ability to respond to selection through phenotypic changes, face reduced chances of survival in new environments (Lande & Shannon, 1996; Merilä & Hendry, 2014). Adaptive potential and species ability to evolve depends on the level of genomic diversity (Day, 2015). While ecological factors are key determinants for invasion success, the role of genetics has been more challenging to demonstrate. Invasions are often characterised by a “genetic paradox”: despite reductions of genetic diversity during population establishment from a limited number of founding individuals, invasive species typically show high capability to adapt to novel conditions that sometimes allows them to outperform native species (Dlugosch *et al*., 2015; Estoup *et al*., 2016). This can be partially explained by reduced environmental pressure, for example resulting from release from natural enemies (Brian & Catford, 2023). On the other hand, admixture of phylogenetically divergent populations coming into contact in the invasive range can restore genetic diversity. Genomics of invasive species can be leveraged to identify populations of origin and invasion routes, and to estimate the timing of invasion and associated demographic bottlenecks, potentially identifying sources creating high levels of genetic diversity in invasive populations. Further, genomics can be employed to assess the role of pre‐existing adaptation on facilitating invasion, and to identify the occurrence of *in situ* adaptations following invasion (North, McGaughran & Jiggins, 2021; Roe *et al*., 2019). Balancing selection can facilitate maintenance of genetic variants important for adaptation to new conditions. New selective pressures can lead to selection of genetic variants acquired either before or after arrival in the invasive range. The latter can occur *via* admixture or introgression from other species or *via* mutations. Indeed, rapid adaptation is proposed to be crucial for the long‐term success of invasive species (Dlugosch & Parker, 2008; Lee, 2002; Rollins *et al*., 2013; Stern & Lee, 2020).

The importance of studying the genetics of biological invasions has been recognised for decades (Baker & Stebbins, 1965; Gray, 1986). During this period, numerous reviews have summarised the mechanisms that are prerequisites for creating and maintaining the levels of genetic diversity allowing for rapid adaptation and successful invasion. Despite many years of study, we still lack a comprehensive summary evaluating most common genetic mechanisms associated with successful invasion across species and environments. Crucial factors facilitating invasion at the level of genetic diversity were reviewed by Bock *et al*. (2015) and Dlugosch *et al*. (2015) before the genomics era. Technological and analytical advancements in the field of genomics have since transformed biological research and contributed significantly to unravelling the evolutionary basis of the success of invasive species (Jaspers *et al*., 2021). The newest reviews referring to technological advances in studying the genomics of invasive species (McGaughran *et al*., 2024; North *et al*., 2021) present the current state of knowledge and future perspectives, but do not synthesise the numerous research findings to resolve the mechanisms involved in species invasions using genomic data. The number of articles utilising genomic methods to pinpoint or suggest different evolutionary mechanisms explaining the successful invasion of different species is growing rapidly.

Here we present a systematic review of studies that have accumulated since 2015 and that applied genomic tools to investigate mechanisms of successful invasions in fungi, plants, insects, and vertebrates. We address hypotheses related to mechanisms of species invasions and evaluate the frequency with which they appear in recent genomics literature. We focus on the main patterns associated with invasions, including changes in genetic diversity at the population level induced by demographic factors such as genetic bottlenecks or gene flow in the invasive range, as well as the evolutionary processes enabling adaptation, such as selection and adaptive introgression that are often related to invasion success. We examine the extent to which different invasion mechanisms have been studied across ecosystems. The objectives of this review include: (*i*) identifying the frequency of molecular footprints of adaptation in invasive populations and investigating whether specific selection events in the native species range are linked to their adaptation in the invasive range; (*ii*) determining the impact of demographic mechanisms that increase the levels of genomic diversity, such as admixture of divergent populations and interspecies hybridisation, in successful invasive populations; (*iii*) determining the impact of demographic mechanisms that reduce the levels of genomic diversity, such as genetic drift and bottlenecks, in invasive populations; (*iv*) identifying how often the levels of genetic diversity have been studied in the context of native populations and invasion routes; (*v*) analysing the genome characteristics associated with invasion success; and (*vi*) unravelling how often studies performed in a genetic context reported non‐genetic factors as responsible for successful invasion.

As a result of this systematic review of articles on genomic diversity of invasive species, we also created a searchable database that can be used by future investigators.

## II. METHODS

Our review followed a systematic review methodology according to the Collaboration for Environmental Evidence guidelines https://environmentalevidence.org/standards-table/. Published studies were identified by searching the *Web of Science* v.5.22.1 (https://www.webofscience.com/wos/) database with search years including articles published in 2015–2023. The specific search strings, the criteria for accepting articles and the methodology for extracting information for inclusion in our database, as well as a summary of the search is given as online Supporting Information in Appendix S1. The final collection of articles was assessed by reading the full text. While performing the text screening, we relied on the information and interpretation of results provided by the article authors. Therefore, the information that the genetic diversity was “high” or “low” or whether there was a decrease or increase in genetic diversity is based solely on the interpretation given in each article. Database S1 provides our full data set of all included articles, and the information collected from each article. Table 1 provides a glossary explaining population genetics terms in relation to species invasions.

**Table 1:** Glossary of population genetics terms used herein in relation to species invasions.

Term	Definition
Adaptive introgression	The transfer by introgression of relatively small genomic regions from a donor species that have positive fitness consequences in the recipient species. Contact between related, but previously isolated, species that occurs after invasion outside a species' natural range may result in adaptive introgression increasing the adaptive potential of invasive species.
Additive genetic variance	The total effect of loci measurably contributing to the trait.
Admixture	The process of mixing genetically distinct populations resulting in exchange of genetic variants between those populations. Multiple introductions of divergent invasive populations may result in increased overall genetic diversity.
Balancing selection	Natural selection that maintains genetic variation within a population by preserving multiple alleles at a particular locus. Refers to any type of selection that maintains genetic variance in a population, such as frequency‐dependent selection, temporally or spatially fluctuating selection, and overdominance. Under appropriate conditions, temporally fluctuating selection could promote the accumulation and maintenance of genetic variation in the native range allowing faster adaptation in the invasive range.
Bottleneck	A sudden reduction in population size (demographic bottleneck), resulting in a reduction of genetic diversity (genetic bottleneck), occurring over one or few generations or a longer period. Bottleneck can act as a selective force when deleterious alleles are stochastically removed and the prevalence of adaptive alleles increases.
Bridgehead effect	Occurs when particular invasive population/populations are invasion sources for other invasive populations due to secondary introductions.
Directional selection	Natural selection where certain alleles are promoted, leading to diminishing genetic diversity in particular loci. Directional selection is expected to occur during population expansion into new environments as a result of response to novel environmental variables.
Genetic drift	Random change in allele frequencies following the colonization of a new range resulting in the loss of genetic diversity and random shifts in allele frequencies.
Effective population size	The number of individuals breeding in the population. Quantifies the magnitude of genetic drift and inbreeding in real‐world populations. Range expansions during invasions should result reduced effective population size (*N*_*e*_) due to strong effects of genetic drift.
Founder event	Establishment of a population from a small number of individuals resulting in a loss of genetic diversity and change in allele frequencies relative to the source population.
Genetic redundancy	A situation in which two or more genes encode a particular function. In the case of a deleterious mutation in one of those genes, the mutation effect on fitness is lower than expected.
Hard selective sweep	A pattern of genetic diversity when a single adaptive haplotype rises to high population frequency. Usually a result of a major effect mutation that arises on a single haplotype in a population and ultimately reaches fixation. As a consequence, the expected haplotype homozygosity surrounding the selected site is high.
Hybridization	Mating of individuals from different species or genetically distinct populations, leading to offspring with mixed genetic ancestries.
Introgression	An effect of hybridization when a foreign variant is permanently incorporated in the local gene pool through back‐crossing.
Mitogenome	The mitochondrial genome.
Polygenic adaptation	A type of adaptation where many loci, each of small effect, contribute to the phenotype. Response to selection is caused by small frequency shifts of many alleles.
Selective sweep	Increase in frequency of favoured alleles caused by selection.
Soft selective sweep	A pattern of genetic diversity when multiple alleles at the same locus become favoured and their frequency increases. Can originate from standing genetic variation that becomes beneficial in a changing environment, or new recurrent *de novo* adaptive mutations. In the case of invasive populations, genetic variation carried from native range may become beneficial in the invasive range resulting in a soft sweep. Soft sweeps do not reduce genetic diversity around the beneficial mutation to the same extent as hard sweeps and may resemble patterns expected under neutrality.
Standing genetic variation	The genetic variation present in the population, as such constituting a source of variation on which selection can act more rapidly than on new mutations. In the case of invasive species is usually defined as genetic variation transferred from the native range.

## III. SUMMARY OF COLLECTED STUDIES

### (1) Taxonomy

**Fig. 1:**
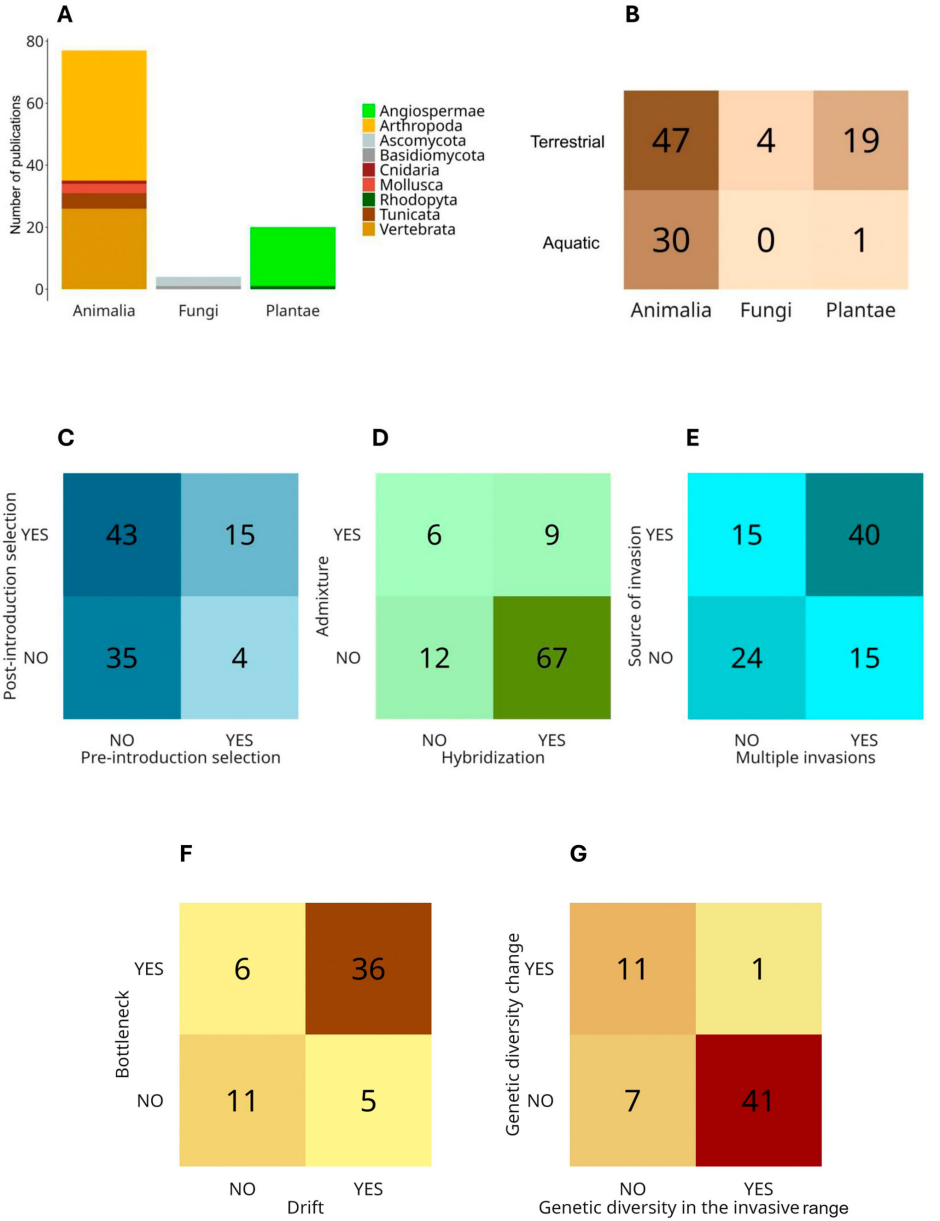
(A) Number of publications identified by our search terms according to kingdom. (B) Number of publications in different kingdoms in terrestrial *versus* aquatic habitats. (C) Publications studying selection occurring pre‐ and post‐introduction into the invasive range. (D) Publications studying admixture and hybridisation. (E) Publications studying source of invasion and the number of invasions. (F) Publications measuring genetic bottlenecks and/or genetic drift. (G) Publications assessing the change in genomic diversity between native and invasive ranges and/or quantifying the level of genomic diversity. Genomic diversity is categorised as: maintained = moderate decrease, no change, or increase in comparison with native populations; or decreased = marked decrease in comparison with native populations. Genomic diversity in the invasive range is categorised as: high = reported as high in the invasive range; or low = reported as low in the invasive range. Classification as high or low and maintained or decreased is based solely on the interpretation of the authors of each study.

Our search retrieved studies of species belonging to the kingdoms Animalia, Plantae and Fungi, with the highest proportion being animal species (Fig. 1A). Among these, the most represented phyla were Vertebrata and Arthropoda. Plant species were less represented, while only four studies focused on Fungi. Terrestrial animals were studied most frequently, followed by aquatic animals and terrestrial plants (Fig. 1B).

### (2) Types of studies

The search resulted in a database of 120 papers further classified into 101 “population studies” (i.e. analysing genetic diversity of invasive and/or native populations) and 19 genome, transcriptome, or genome and transcriptome analyses, which examined single/several genomes or transcriptomes of invasive species in the context of invasion. Twenty‐four population studies also included genome/transcriptome analyses. Among the population studies, the most frequent topics investigated were selection processes in the invasive range and demographic events describing invasion (invasion sources and routes; number of invasions). Admixture between different invasive populations was more frequently a focus of study than hybridisation with other species and the majority of research related the analysis to native source populations (Fig. 1C–E).

### (3) Applied methods

**Fig. 2:**
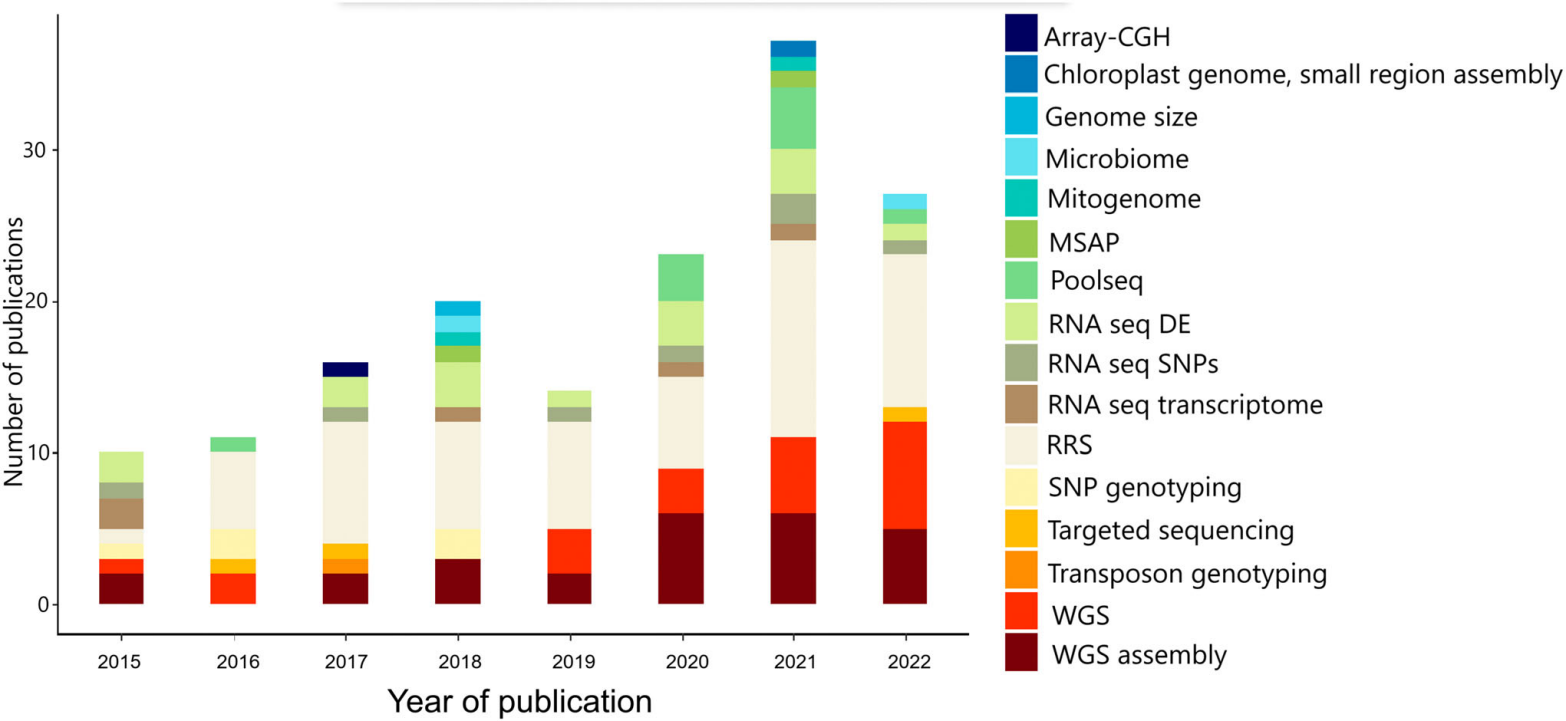
Genomic methods applied in the studies included in our database for the period 2015–2022. CGH, comparative genetic hybridisation; DE, differential gene expression; MSAP, methylation sensitive amplified polymorphisms; RRS, reduced representation sequencing; SNP, single nucleotide polymorphism; WGS, whole genome sequencing.

The sequencing methods used in a study determine the analytical approach that can be applied and the types of questions that can be addressed. Nearly half of the selected studies (57/120 studies) were based on reduced representation sequencing (RRS) methods. In population studies, 21/120 studies conducted whole genome sequencing (WGS) and nine/120 pooled sequencing (PoolSeq), with the remaining studies using less‐common methods. Only 26/120 studies leveraged at least one whole genome sequence assembly, and five studies used transcriptome assembly for their analyses. The number of studies employing genomic methods has changed over time with a recent increase in the number of studies using WGS and RRS (Fig. 2).

## IV. GENOMIC DIVERSITY CHANGE AND THE LEVEL OF DIVERSITY

### (1) The level of genetic diversity: does genetic diversity decrease in invasive populations?

Previous reviews found contrasting trends in the levels of genetic diversity in invasive populations. These surveys analysing the genetic diversity of wild populations in an invasion context were performed before the genomic era, and therefore analysed studies mostly investigating a small number of traditional, presumably neutral markers (allozymes, microsatellites, mitochondrial, etc.). Two such analyses found that 69% of invasive plants (Bossdorf *et al*., 2005) and 63% of aquatic invaders (Roman & Darling, 2007) had levels of genetic diversity equal to or even greater than native populations. By contrast, Uller & Leimu (2011) reported moderate reductions in genetic diversity for the majority of invasive species. Using a limited number of neutral markers may lead to inadequate estimation of genetic diversity or missing information from ecologically relevant regions of the genome. A comprehensive assessment of genomic diversity is the most representative predictor of a species' evolutionary potential.

Out of the 101 population studies retrieved by our search, 62 analysed both native and invasive ranges. A comparison of genetic diversity levels in native and invasive ranges was provided in 51 studies out of these 62, but a statistical comparison of genomic diversity levels between source and invasive populations often was missing. There was, however, a clear pattern that statements of a reduction in genomic diversity in invasive populations are infrequent. We subdivided the studies into those stating that invasive populations show decreased genomic diversity (19/101) and those declaring that genetic diversity was maintained (here we combined articles reporting an increase, stable level or minor decrease of genetic diversity as stated by the authors; 43/101; Fig. 1G). In the remaining studies (39/101), no comparison of diversity levels between native and invasive ranges was performed, but in the majority of these, the level of diversity was indicated as high with only eight studies reporting low diversity in invasive populations. These findings are in line with previous results showing that the “genetic paradox” is not a general phenomenon in invasive species. Many studies did not assess genomic diversity levels at different invasion stages, with the majority performing sampling of native and invasive ranges at one time‐point only. Sampling of genomic diversity across a time series in invasive ranges would allow assessment of diversity loss as a result of population establishment from a limited number of founders and to what extent it can be restored with time. Such data would also allow researchers to assess the speed of evolutionary changes at different invasion stages.

### (2) The role of demographic factors: do invasive populations experience bottleneck/drift?

**Fig. 3:**
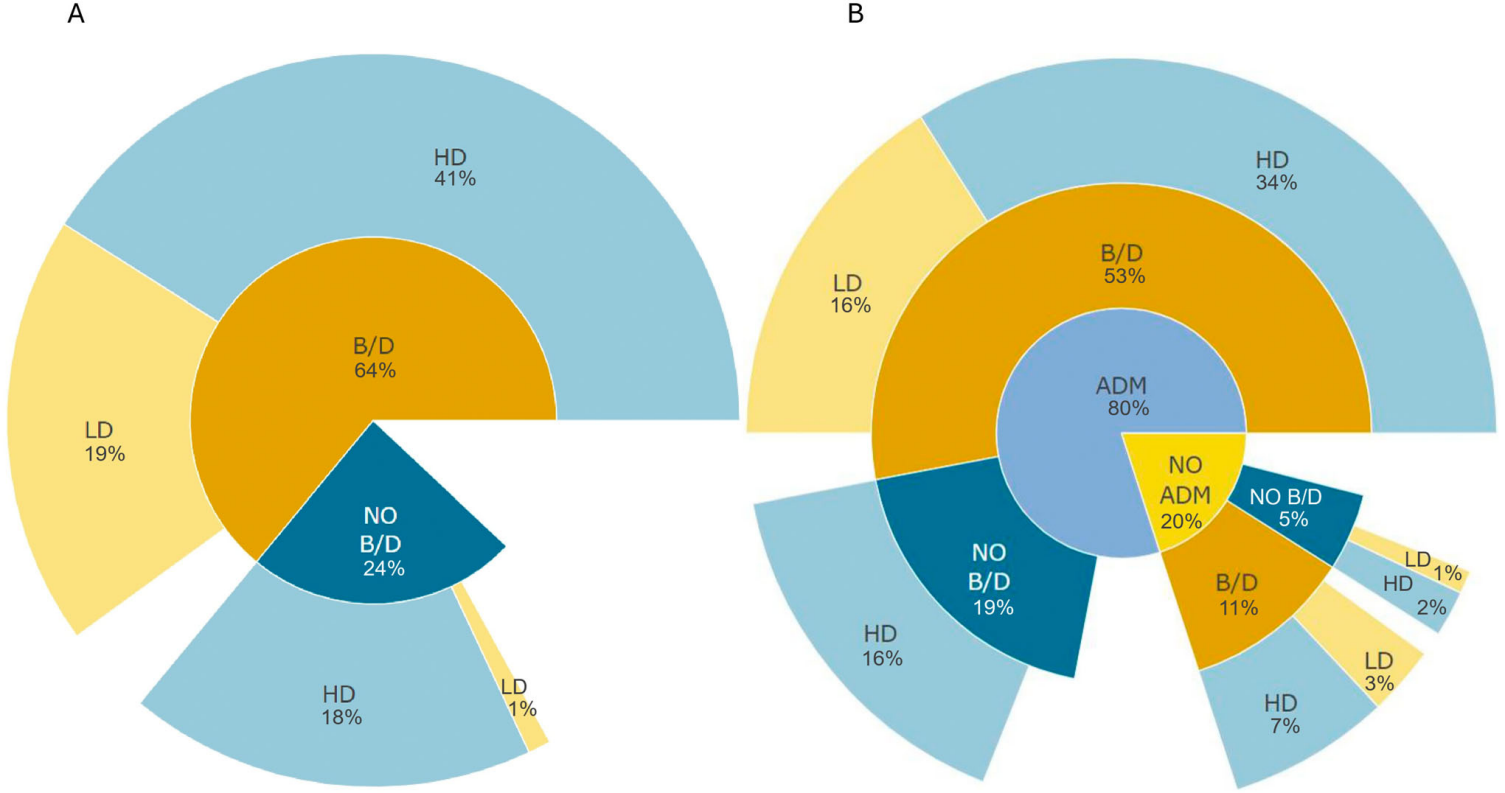
Demographic factors shaping genetic diversity in invasive populations: (A) the percentage of articles detecting the presence of genetic bottleneck and/or genetic drift (both or one of the mechanisms was assessed in the article) or their absence (none of the mechanisms was confirmed in the article), and the percentage of articles where genetic diversity of invasive populations was assessed by the authors of each article as high or low; (B) the percentage of articles where admixture was reported or not, the percentage of articles detecting the presence of genetic bottleneck and/or genetic drift or not and the percentage of articles where genetic diversity of invasive populations was assessed by the article authors as high or low. Empty regions reflect a lack of information in the analysed articles. ADM, presence of admixture; B/D, presence of genetic bottleneck and/or genetic drift; HD, high level of genetic diversity in invasive populations; LD, low level of genetic diversity in invasive populations; NO ADM, absence of admixture; NO B/D, absence of genetic bottleneck and genetic drift.

Despite the high levels of genetic diversity reported in the majority of studies (Fig. 1G), both genetic drift (detected in 47 of 101 studies), and bottlenecks (56 of 101 studies) were reported relatively frequently (Figs 1F, 3). Our approach relied on identifying specific key words when screening the text, and we note that some authors used one term but not the other. In most cases high levels of genetic diversity were restored by admixture between populations (Fig. 3B), recurrent invasions or the presence of bridgehead populations or introgression from other species. In several studies a lack of bottleneck signal was attributed to high propagule pressure during establishment in the invasive range (Chen *et al*., 2021a; Goubert *et al*., 2017) or to interspecific hybridisation before invasion (Burford Reiskind *et al*., 2019; Popovic *et al*., 2021). Bottlenecks can increase variation for quantitative traits by changing the relative magnitude of the genetic variances component, leading in some cases to an increase and allowing rapid evolutionary change in newly established populations (Turelli & Barton, 2006). Deleterious variants that have a higher probability of exposure in homozygotes after a population bottleneck can be purged more effectively from the population (Rius *et al*., 2015). It has been suggested that bottlenecks can also act to increase additive genetic variance for traits with a non‐additive genetic basis (Mularo, Bernal & DeWoody, 2022). Increased genetic variance resulting from a bottleneck was suggested in *Agarophyton vermiculophyllum* (Ohmi) populations (Flanagan *et al*., 2021) that rapidly evolved greater tolerance to acute high temperatures and low salinities in the invasive range (Sotka *et al*., 2018).

Although the occurrence of bottlenecks is frequently reported, the studies in our database lack time‐series estimates that would enable precise detection of the timing of the event, the quantification of the impact of specific admixture events, and the interplay between these two factors.

### (3) The role of admixture and introgression: do invasive populations experience gene flow?

**Fig. 4:**
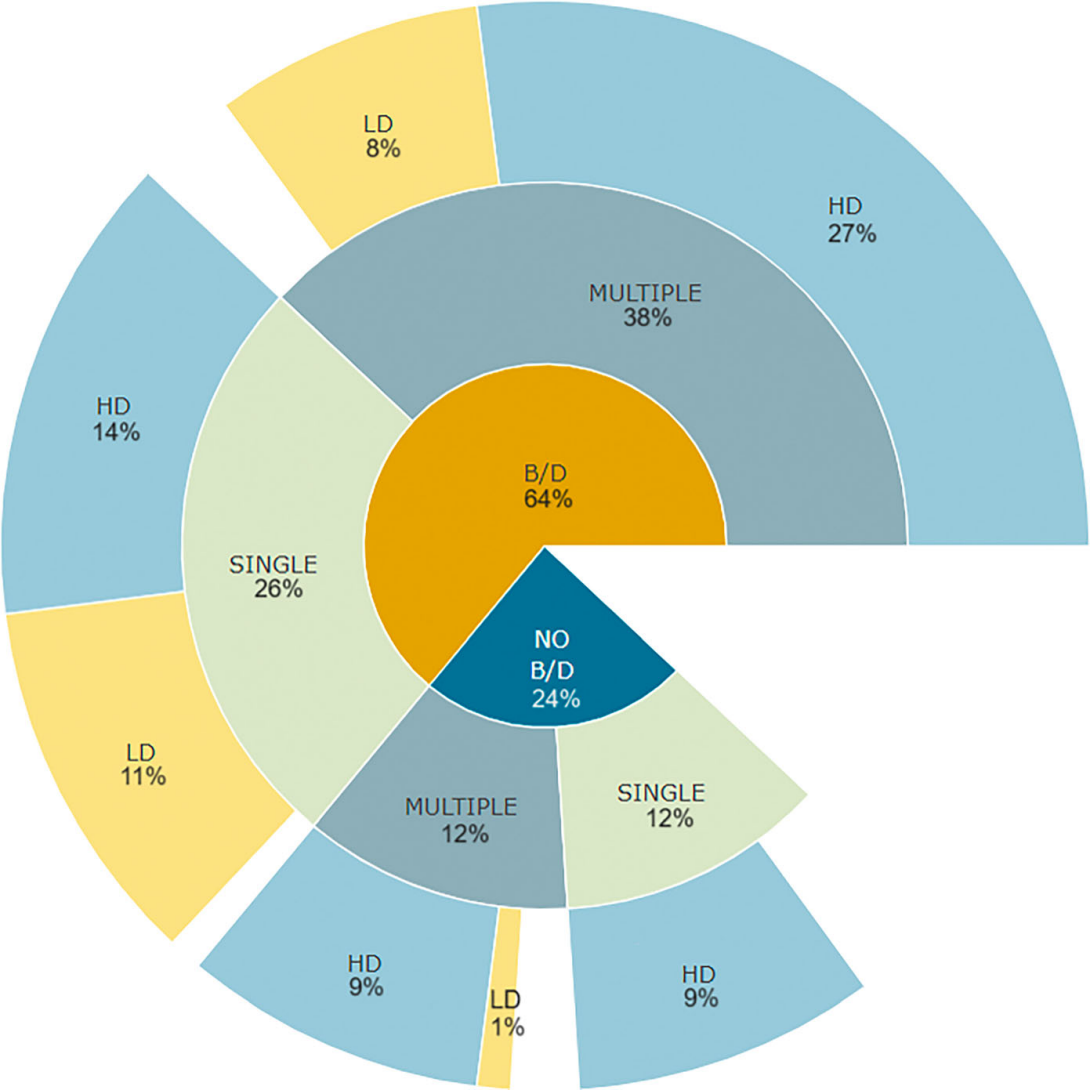
Demographic factors shaping genetic diversity in invasive populations. Percentage of articles detecting the presence of genetic bottleneck and/or genetic drift (both or one of the mechanisms was assessed in the article) or its absence (none of the mechanisms was confirmed in the article), the percentage of articles where either single or multiple introductions were detected and the percentage of articles where genetic diversity of invasive populations was assessed by the authors of each article as high or low. Empty regions reflect a lack of information in the analysed articles. B/D, presence of genetic bottleneck and/or genetic drift; HD, high level of genetic diversity in invasive populations; LD, low level of genetic diversity in invasive populations; MULTIPLE, detection of more than one event of introduction; NO B/D, absence of genetic bottleneck and genetic drift; SINGLE, detection of one introduction.

Admixture between genetically divergent populations increases overall diversity and may foster genetic interactions among previously isolated alleles, allowing higher fitness of admixed populations and increasing the adaptive evolution of novel genotypes (Wagner *et al*., 2017). The majority (61/80 studies studying admixture) demonstrated ongoing admixture at different stages of the invasion leading to increased genetic diversity (Fig. 3B). Only 20 out of all 101 population studies of studies reported no admixture and 19 out of 80 studies studying admixture reported a lack of significant change in the level of genomic diversity as an effect of admixture. Among these 19, high levels of diversity resulted from interspecific introgression or hybridisation in the native range. Therefore, an important finding of our survey is that admixture of invasive populations that substantially mitigates the effects of genetic drift and bottlenecks is extremely widespread (Figs 3B, 4). It appears that admixture of multiple invasive populations and, to some extent, interspecific introgression are the main mechanisms maintaining and increasing genomic diversity of invasive populations. Some studies have questioned the association between admixture and invasion success and its contribution to increased fitness of invaders (Chapple *et al*., 2013; Dutech *et al*., 2012; Wolfe, Blair & Penna, 2007). Nevertheless, the great majority of our surveyed studies identify admixture between populations as a significant process increasing genetic diversity and invasion success. One of the major unresolved questions in invasion genomics is whether, and to what extent, admixture is a cause or a result of successful establishment of invasive species, in particular when multiple invasions occur. These scenarios are non‐mutually exclusive, but with careful study design or temporal sampling, genomics can be used to answer such questions. For example, population genomics can be used to infer the timing of admixture events, selective sweeps of introgressed genes, and demographic expansions, while experimental approaches, such as common garden experiments, can be used to compare the fitness of admixed *versus* non‐admixed invaders.

### (4) The role of mutation load on invasion success: do invasive populations carry a mutation load?

Further evidence for the lack of a “genetic paradox” is the absence of mutational load in invasive populations. Reduced effective population size during invasions may lead to inbreeding (Fauvergue *et al*., 2012), which increases the homozygosity of segregating deleterious recessive alleles (i.e. genetic load) leading to a loss of fitness (i.e. inbreeding depression; Charlesworth & Willis, 2009). Additionally, in small populations, the frequency of deleterious alleles may increase stochastically (i.e. drift load), compromising fitness (Willi, Griffin & Van Buskirk, 2013). However, the genetic load in newly established invasive populations can be rapidly reduced by selection (Lacy & Ballou, 1998), admixture between populations from multiple introductions, or hybridisation in the new range. Rare deleterious alleles, usually present at low frequencies, can also be lost as a result of bottleneck upon introduction (Nei, Maruyama & Chakraborty, 1975). In fact, only two studies in our database suggested the possibility of genetic load in the genomic data. An analysis of the genomes of five Asteraceae species showed a greater proportion of deleterious alleles in the genome in the invasive range for one species, while the remaining species had a higher number of deleterious alleles in the native range (Hodgins *et al*., 2015). The other study, analysing genome‐wide diversity of two hybridising invasive species *Cakile edentula* and *Cakile maritima* predicted a greater fixation rate of weakly deleterious alleles in *C. edentula* due to its higher level of inbreeding (Rosinger *et al*., 2021). The low number of recovered studies investigating genetic load reflects the rarity of whole‐genome sequencing of large invasive populations coupled with fitness measurements.

## V. GENOMIC FOOTPRINT OF ADAPTATION

### (1) Preintroduction adaptation

One important consideration when analysing invasion success is the level of standing genetic variation available in invasive populations that can be leveraged during adaptation to novel habitats.

A fluctuating environment in the native range promotes the maintenance of multiple genetic variants (Lee & Gelembiuk, 2008). Diversity can in turn serve as a catalyst for selection in response to novel habitat conditions (Stern & Lee, 2020; Vera, Díez‐del‐Molino & García‐Marín, 2016). Studies of genes shaped by balancing selection in both native and invasive habitats suggest that the retention of multiple variants of specific genes may be more important for survival of native and invasive populations than generally high levels of diversity across the genome (Vera *et al*., 2016). This was shown to be the case for immune genes in native and invasive populations of the common raccoon (*Procyon lotor*) (Konopiński, Fijarczyk & Biedrzycka, 2023) where maintaining multiple variants provided protection from various pathogens.

High levels of adaptive genetic diversity in the invasive range can also be maintained by diversifying selection acting on the same loci as in the native range (Burford Reiskind *et al*., 2019; Goubert *et al*., 2017; Krzemińska *et al*., 2018). This special case of diversifying selection occurs when environmentally important traits have a polygenic background. Temperature adaptation is a well‐documented polygenic trait and it can operate through different combinations of loci. Differentiated frequencies of “cold‐adapted” variants favoured at lower temperatures and “warm‐adapted” variants at higher temperatures reflect adaptation over a relatively short timescale and show that genetic variation that evolved in the native habitat is a primary factor allowing rapid adaptation across a thermal gradient in a new habitat (Barghi *et al*., 2019; Krehenwinkel, Rödder & Tautz, 2015; Popovic & Riginos, 2020; Yang *et al*., 2022). This suggests that invasion bottlenecks have a limited capacity to attenuate polygenic traits. The genetic redundancy resulting from polygenic adaptation may be an important mechanism facilitating adaptation in invasive species (Láruson, Yeaman & Lotterhos, 2020). Among population studies (*N* = 101), we identified 62% that included both native and invasive populations, 35% that studied only invasive populations, while in three studies only native populations were analysed. Only 15% of population studies focused on selection footprints in both native and invasive populations (Fig. 1C), where mechanisms in native populations were investigated for their role in facilitating adaptation in invasive populations. Examples of mechanisms responsible for creating adaptive genomic diversity in native habitats that contributed to the diversity increase upon introduction in the invasive ranges are listed in Table 2.

**Table 2:** Examples of different selection mechanisms acting in the native range that may contribute to diversity increase upon invasion.

		Examples	References
Mechanisms promoting maintenance of high levels of diversity	Balancing selection	*Balancing selection in the native habitat that is driven by fluctuating environmental conditions and followed by directional selection in the invasive range*. In the copepod *Eurytemora affinis*, salinity fluctuations in the native environment and short, overlapping generations foster balancing selection (Stern & Lee, 2020). Directional selection occurred in the invasive populations at ion transport genes, which were under balancing selection in the native habitat. *Balancing selection in both the native and invasive range, maintaining multiple variants of specific genes*. In Eastern mosquitofish, *Gambusia holbrooki* (Vera *et al*., 2016) genes related to reproduction, growth and development were under balancing selection in both the native and invasive range despite a clear reduction in genetic diversity. In common raccoon (Konopiński *et al*., 2023) single nucleotide polymorphisms (SNPs) located in several immune genes were maintained under balancing selection in native and invasive range.	Stern & Lee (2020); Konopiński *et al*. (2023); Vera *et al*. (2016); Mittan‐Moreau *et al*. (2022)
	Sharing SGV (standing genetic variation) and gene reuse	*Rapid and repeatable phenotypic and genomic adaptation in the native and invasive habitats* of common ragweed *Ambrosia artemisiifolia*. Candidate adaptation loci overlapped between ranges. The consistency in habitat characteristics between native and invasive ranges suggests that shared standing genetic variation could increase the probability of gene reuse during rapid local adaptation (van Boheemen & Hodgins, 2020).	van Boheemen & Hodgins (2020)
	Polygenicity	*Polygenic adaptation to temperature* in potato ground beetle (Yang *et al*., 2022) and European green crab (Tepolt & Palumbi, 2020). High temperature tolerance in European green crab (*Carcinus maenas*) can explain its worldwide invasiveness. Differentiated frequencies of “cold‐adapted” and “warm‐adapted” variants in different temperatures reflect adaptation on a short timescale and show that genetic variation that evolved in native habitat is a primary factor allowing rapid adaptation across a thermal gradient in its new habitat.	Yang *et al*. (2022); Tepolt & Palumbi (2020)
Mechanisms promoting specific variants: diversifying selection		*Differential diversifying selection acting at the same loci in native and invasive ranges*. Found in genes putatively related to boldness and anxiety behaviours, reproduction, and food intake suppression in the red lionfish (*Pterois volitans*) (Burford Reiskind *et al*., 2019). In Asian tiger mosquito (*Aedes albopictus*) reported in genes related to lipid metabolism and juvenile hormone‐binding protein (Goubert *et al*., 2017). *Diversifying selection in the native range followed by directional selection in the invasive range*. In house crow (*Corvus splendens*) reported in genes related to environmental adaptation.	Burford Reiskind *et al*. (2019); Lin *et al*. (2017); Konopiński *et al*. (2023); Goubert *et al*. (2017); Tepolt & Palumbi (2020); Krzemińska *et al*. (2018)
Unspecified mechanism		*Allele frequency change between native and introduced populations*. Studies do not specify the selection footprint, only report specific outlier loci in both ranges.	Merel *et al*. (2021); Narum *et al*. (2017); Flanagan *et al*. (2021); Helliwell *et al*. (2018)

Although the importance of adaptation in the native habitat has been acknowledged by a number of studies, the exact causes of its presence are sometimes difficult to identify. For example, within the native and invasive ranges of the house crow (*Corvus splendens*), signatures of natural selection were detected in the mitogenome, but these were associated both with lineage diversification and recent adaptation to environmental conditions (Krzemińska *et al*., 2018). In the invasive Asian tiger mosquito (*Aedes albopictus*), adaptive genetic and morphometric variation observed along the climatic gradient of the native range suggests that colonisation of northern latitudes promoted adaptation to cold environments prior to its worldwide invasion (Sherpa, Blum & Després, 2019b). Another genomic study on *A. albopictus* invasion (Goubert *et al*., 2017) found selection footprints both in native and invasive populations, but it was impossible to delineate whether these were related to adaptations prior to invasion or were a recent shift in allele frequencies that reflects adaptations to cooler invasive habitats.

Adaptations that evolve in the native range are often affected by genetic drift upon establishment of invasive populations. A joint effect of demographic and selective forces affecting levels of genetic diversity and allele frequency change was observed in 9 out of 14 articles studying invasive and native populations where both selection and genomic drift were identified. In all but one of those cases, loss of genetic diversity was counteracted by gene flow between differentiated source populations in the invasive range. For example, in Chinook salmon (*Oncorhynchus tshawytscha*) (Narum *et al*., 2017), exposure to novel pathogens in native populations resulted in selection on immune‐related single nucleotide polymorphisms (SNPs) but the observed genomic pattern was then erased by genetic drift and gene exchange between different source populations in the introduced environments. Despite clear evidence of the presence of adaptive alleles in the native range and transmission of this genetic diversity into the invasive range, associations that should arise in the invasive range are sometimes not observed, suggesting the loss of adaptive capacity. For example, populations of sweet vernal grass (*Anthoxanthum odoratum*) were adapted to soil aluminium toxicity in their native range, but not invasive range, despite the presence of specific allelic variants (Gould & Gerber, 2016). This lack of association could be explained by genetic linkage or genetic background effects that may be different in the native and introduced ranges.

Findings from the studies linking pre‐introduction adaptation in the native range with increased invasion ability illustrate the significance of prior adaptation for invasion success in new habitats. However, the number of such studies is limited and there are several reasons why we lack evidence. First, to detect such associations, the identification of source populations of an invasion is necessary. Further, both native and invasive populations must be sampled and sequenced. Precise demographic inferences, such as population substructure in both ranges, changes in effective population sizes, and levels of gene flow in both native and invasive populations also need to be estimated (Teshima, Coop & Przeworski, 2006). Additionally, the appropriate statistical measures that take into account demographic events must be applied, especially in the case of balancing selection. When detecting multiple variants at intermediate frequencies at the population level, the results can be confounded by demographic processes. Moreover, the effects of selection, both in native and invasive populations, ideally should be experimentally tested or associated with environmental variables to demonstrate adaptation.

### (2) Adaptation in the invasive range

Once a species has invaded, adaptation in the invasive habitat enhances its ability to persist and thrive by rapidly shifting its ecological niche to match the new environment better (Sultan *et al*., 2013; Vandepitte *et al*., 2014). Rapid acclimation to the new ecological niche can be realised by phenotypic plasticity (Promy, Newberry & Gulisija, 2023; Uller & Leimu, 2011) or direct genetic changes altering functional diversity resulting from natural selection. Here, we focus on genomic mechanisms enabling adaptation. We analyse sources of variation and processes creating genomic diversity, different selection footprints, and signatures of recent selection.

#### (a) *Sources of genetic diversity: standing genetic variation,* de novo *mutations and presence of selective sweeps*

Evolution requires genetic diversity (Clarke, 1979). Selection in the invasive range may act on new mutations or on standing genetic variation (Barrett & Schluter, 2008), leaving a different pattern in the genome. A novel, large‐effect mutation that arises on a single haplotype in a population and ultimately reaches fixation creates a pattern of hard selective sweep. Soft sweeps generally refer to scenarios in which multiple haplotypes carry a beneficial variant that was present in the native range as standing genetic variation (Hernández *et al*., 2011). To date, mounting evidence indicates that although hard selective sweeps are easier to detect in the genome than soft selective sweeps, they might not be the dominant mode of adaptation in many species. Further, identifying the relative contribution of standing *versus de novo* variation to rapid adaptation of invasive species is difficult, as high genomic marker density and reference genome contiguity are required for the identification of *de novo* variation. The degree to which beneficial *de novo* mutations can impact invasion success over the short timescale of invasion is also unclear (Pélissié *et al*., 2018). In our search we did not locate any study that clearly confirmed the presence of adaptive *de novo* genetic diversity that arose in the invasive range and allowed adaptation there. In four studies we found a suggestion that adaptive changes can have a *de novo* origin (Kotsakiozi *et al*., 2017; Lin *et al*., 2017; Mérel *et al*., 2021; Sherpa *et al*., 2019c), but none of these studies performed a formal test proving the origin of this diversity. Standing genetic variation as a source of adaptive variants was reported in 24 out of 101 studies, the remaining studies did not specify the source of variation. In nine studies, homozygosity runs or regions with linkage disequilibrium (LD) were interpreted as a footprint of selective sweeps (Wu *et al*., 2019; Lin *et al*., 2017; Krehenwinkel *et al*., 2015; Chen *et al*., 2021b; Hübner *et al*., 2022; Liu *et al*., 2022; Wegner, Lokmer & John, 2020; Konorov *et al*., 2021; Yoshida *et al*., 2016), but without a clear distinction between soft or hard sweep. It is important to note that the length of the selective sweep footprint left on genomic regions depends strongly on recombination rate (Garud, 2023) which can greatly narrow down the hitchhiking effect (Fay & Wu, 2000).

#### (b) Selection footprints in the invasive range

Adaptation of species in the invasive range results from different types of selection, including directional, divergent, or parallel selection. In cases where multiple genetic variants provide better adaptation to new environments, balancing selection occurs in the invasive range. Conversely, if variants brought into a new range confer lower fitness in the novel conditions, negative or purifying selection is expected.

The most common result reported as a proof of selection in the invasive range was a change in allele frequency (outlier loci) between native and introduced populations (7 out of 36 studies). These studies neither interpreted further the type of selection nor related outliers to specific selective pressures. Our survey did not find evidence that balancing or diversifying selection occurring in the native range (Lee, 2016) is the common driver of directional selection in the invasive range. Nevertheless it is important to note that although balancing selection is increasingly acknowledged as a widespread mechanism creating genetic diversity within populations, its detection is challenging. For genomic data, high SNP densities are needed as signatures of balancing selection are often much narrower than for other selection types (Bitarello *et al*., 2023). The most prominent examples of the role of balancing selection in the native range for the success of invasive species show a repetitive response to selection acting on the same loci in native and invasive populations [*Eurytemora affinis* (Stern & Lee, 2020), *Gambusia holbrooki* (Vera *et al*., 2016)]. Similarly, in the invasive European green crab (*Carcinus maenas*), adaptation to wide temperature spectra enabled successful adaptation in the invasive range (Tepolt & Palumbi, 2020). Five studies reported balancing selection in the invasive range, and 12 out of 101 studies reported only the presence of outlier loci between native and invasive populations, without interpreting the type of selection. Our results show that favourable genetic variants brought from native ranges are often a source of rapid adaptation to novel habitat characteristics.

The genomic pattern left by selection in an invasive population linked to environmental selection agents can provide confirmation of rapidly occurring adaptations (Gautier, 2015). However, statistical associations between specific gene variants and environmental characteristics are relatively rare in our database. In only 12 out of 60 studies were such associations reported, offering direct evidence for adaptation to environmental variables in the new range. Gene–environment analyses (GEAs) require gathering environmental data and sampling a relevant number of individuals. Additionally, large, repetitive genomes, and high levels of gene flow between diverse populations affecting population structure and causing a decrease in locally adaptive variants *via* genetic swamping, make GEAs challenging. The small number of such studies reveals a significant gap in research on adaptation in invasive species and indicates that defining relevant environmental characteristics or traits relevant for invasion success remains challenging. More examples documenting such associations will improve our understanding of the genetic architecture of local adaptation.

**Fig. 5:**
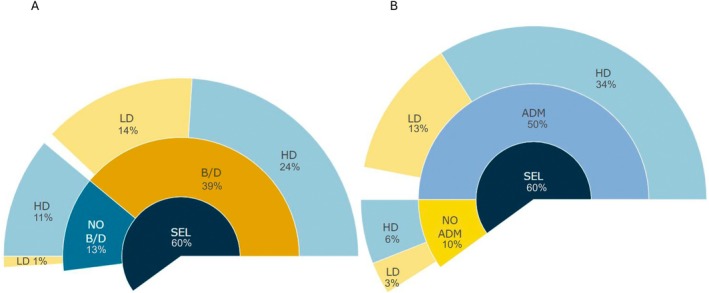
Sunburst charts displaying the frequency of demographic factors affecting genomic diversity in articles where selection was detected: (A) the percentage of articles where selection was detected, the percentage of articles detecting the presence of genetic bottleneck and/or genetic drift (both or one of the mechanisms was assessed in the article) or absence (none of the mechanisms was confirmed in the article), and the percentage of articles where genetic diversity of invasive populations was assessed by the authors of each article as high or low; and (B) the percentage of articles where selection was detected, the percentage of articles where admixture was reported or not and the percentage of articles where genetic diversity of invasive populations was assessed as high or low. Empty regions reflect the lack of information in the analysed articles. ADM, presence of admixture; B/D, presence of genetic bottleneck and/or genetic drift; HD, high level of genetic diversity in invasive populations; LD, low level of genetic diversity in invasive populations; NO ADM, absence of admixture; NO B/D, absence of genetic bottleneck and genetic drift; SEL, presence of selection in invasive range.

Regardless of the type of selection footprint detected in invasive populations, the majority of studies report the joint action of selection and bottleneck/genetic drift and high or stable levels of genetic diversity (Fig. 5A).

#### (c) Factors creating genomic diversity in populations showing adaptive changes in the invasive range

High standing genomic diversity is expected to be the primary cause of adaptive potential in invasive species (Tepolt, 2015), both for terrestrial and aquatic environments. We therefore evaluated the levels of genomic diversity in studies where adaptive selection was detected in the invasive populations (60% of 101 population studies, Fig. 5A). Almost half of the studies that detected selection in the invasive range did not show a reduction in genetic diversity. An increase (e.g. Errbii *et al*., 2021; Bieker *et al*., 2022; Wellband *et al*., 2018), no change (e.g. Yoshida *et al*., 2016; Popovic *et al*., 2021; Ni *et al*., 2018), or only a moderate decrease in genetic diversity in the invasive range (e.g. Hübner *et al*., 2022; Yin *et al*., 2021) was reported in 26 out of the 60 studies. For moderate reductions of diversity, the authors often argued that the observed reduction did not affect the species' ability to undergo rapid adaptive evolutionary change. Only 11 out of 60 studies reported a decrease in genomic diversity of invasive populations (Fig. 1G). Using the categorisation of genetic diversity in the invasive range, only 11 of the 60 studies reported low diversity (Fig. 1G) and associated it with genetic bottlenecks upon introduction.

Most studies 39/60 report genetic drift and/or bottleneck as a force shaping genetic diversity in invasive populations (see Section IV.2). In some cases, reduced genetic diversity of invasive populations was reinforced by both genetic drift/bottleneck and diversifying, directional or purifying selection (Wu *et al*., 2019; Pérez‐Portela *et al*., 2018; Vera *et al*., 2016; Sherpa *et al*., 2018; Skrede *et al*., 2021; Selechnik *et al*., 2019; Tepolt *et al*., 2022; Mittan‐Moreau *et al*., 2022; Chen *et al*., 2021c). In the invasive brown rat, *Rattus norvegicus* in China (Chen *et al*., 2021c), a decrease in genetic diversity was caused both by genetic drift and directional selection in loci related to lipid metabolism and immunity in the invasive range. The authors underlined the role of bottlenecks and genetic drift in facilitating purifying selection resulting in more effective elimination of deleterious alleles due to non‐random mating in small populations. In the invasive European green crab, *Carcinus maenas*, successful invasion along the northeast Pacific coastline occurred despite low levels of genetic diversity (Tepolt *et al*., 2022). In this case, rapid cold adaptation acted through a handful of linked SNPs under balancing selection. The authors showed that variation originated from a single inversion polymorphism before the introduction into the invasive range. In fall webworm, *Hyphantria cunea*, selective sweeps and excess of non‐synonymous mutations in gustatory receptors as well as expansion in gustatory receptor genes were found to be associated with novel nutrition sources despite strong bottleneck (Wu *et al*., 2019). These examples show that while high genetic diversity can foster invasive potential, it is not a prerequisite for successful invasion.

The most common phenomenon contributing to an increase in levels of genomic diversity of invasive populations under selection was gene flow within the invasive range, with a majority (36/60) of studies identifying admixture between invasive populations as a primary factor increasing the genomic diversity of invasive populations (Section IV.3). Only 10 out of 60 studies reported no admixture, and 14 out of 60 reported a lack of significant change in genomic diversity. Other factors included recurrent invasions and the presence of bridgehead populations (Kramer *et al*., 2023; Sherpa *et al*., 2018) that eventually led to increased genetic variation. For example, the Caribbean invasive populations of the mosquito *Aedes aegypti* (Sherpa *et al*., 2018) showed signs of genetic drift, two bottlenecks and small effective population size. As a result, invasive populations had overall low genetic diversity, but recurrent invasion events eventually resulted in an increase of genetic diversity and potentially allowed for selection at loci related to metabolic breakdown of insecticides.

High levels of genetic diversity are also generated by introgression, either between divergent lineages of invasive species (Errbii *et al*., 2021; Bieker *et al*., 2022), from related native species (Yoshida *et al*., 2016) or between related non‐native species that occurred prior to introduction (Martin *et al*., 2016). Only 16 out of 101 studies investigated inter‐species hybridisation (Fig. 1D). Adaptive introgression was detected in seven studies, where introgression facilitated adaptation by introducing specific genes/variants into invasive species' genomes. Dutch elm disease is caused by three different fungal lineages *Ophiostoma ulmi*, *Ophiostoma novo‐ulmi* subspecies *novo‐ulmi* and *Ophiostoma novo‐ulmi* subspecies *americana*, which differ in pathogenicity and temperature optimum. Gene flow from the more ancient to the more recent invasive lineages resulted in introgression between lineages that impacted fitness‐related traits (Hessenauer *et al*., 2020). Another example is provided by introgression between two species of *Helicoverpa* moths, where widespread introgression from native to invasive species took place, followed by intensive expansion of the invasive *H. armigera*. Interestingly, selection against introgression after the initial expansion, and back‐introgression of an insecticide‐resistant locus to native species, has resulted in the coexistence of two insecticide‐resistant pest species (Valencia‐Montoya *et al*., 2020).

Taken together, the latest genomic studies on rapid adaptation during invasions provide evidence that while genetic drift often occurs as the result of the establishment of new populations outside their natural ranges, any reduction in genetic diversity typically is not severe and often is counteracted by multiple introductions, admixture or interspecific gene flow. A significant and permanent reduction of genetic diversity is rarely detected in invasive populations, and even where it occurs, does not appear to rule out the chances of rapid molecular adaptation and usually does not impact the species' performance in the invasive range. The classical “genetic paradox” of invasive species, where adaptation in the invasive range is observed despite reduction in genetic diversity, seems to be exceptionally rare.

Similar adaptive mechanisms operate in both the native and invasive populations, yet the latter may experience more pronounced or relaxed selection pressures depending on conditions in the invasive range. Heightened selection pressure from novel environmental conditions in the invasive range may impose selection for higher mutation rates (Travis & Travis, 2002). By contrast, release from natural enemies may reduce or eliminate selective forces in invasive populations (Biedrzycka *et al*., 2023). Additionally, the presence of other species or genetically distinct ecotypes of the invasive species may facilitate hybridisation and introgression in the invasive range. It can be argued that, in many cases, increased selection pressures in the invasive habitat could lead to accelerated evolutionary processes compared to the native habitat.

Studies using genomic data to explore molecular adaptation in invasive populations are undergoing a transition from analyses mainly of demographic processes to research aiming to understand the molecular mechanisms driving rapid adaptation in invasive species.

#### (d) Genes under selection

The superior performance of invasive species should imply that they possess adaptations in genes related to those specific biological features. Gene families known to be associated with invasiveness include families involved in chemosensory abilities (Liu *et al*., 2016; Papanicolaou *et al*., 2016; Sparks & Dickens, 2017; Wu *et al*., 2016; Yuan *et al*., 2016), detoxification metabolism (Andersen *et al*., 2017; Grigoraki *et al*., 2017; Nguyen *et al*., 2016; Tian *et al*., 2017), heat shock proteins (HSPs) (Hu, Chen & Li, 2014; Wang *et al*., 2017) and innate immunity (Beckert *et al*., 2016; Vogel, Schmidtberg & Vilcinskas, 2017). Selection processes were commonly detected in the invasive range, and 46 of those 60 articles identified specific genes or DNA regions acting as selection targets (Table 3). However, the majority of studies used only outlier detection and seldom linked outlier loci with specific selection pressures or assigned significance using quantile thresholds, thus some of the reported results might be false positives. Additionally, the inference between sectional and demographic processes should be taken into account when identifying sites under selection (Lotterhos & Whitlock, 2014). Several articles emphasise that finer resolution genotyping (Hübner *et al*., 2022), whole‐genome sequencing, appropriately large sample sizes (Jeffery *et al*., 2017a; van Boheemen & Hodgins, 2020), and the availability of reference genomes for invasive species (Mérel *et al*., 2021; Formenti *et al*., 2022; Theissinger *et al*., 2023) are needed for the identification of genes crucial for adaptation in the invasive range. We reiterate these recommendations here. For the studies in our database, genes under selection are related to various environmental pressures and functions (Table 3). Additional studies in different invasive systems are needed to identify other invasion‐related gene families, and should include comparative genomic studies on gene family expansions and contractions performed on related invasive and non‐invasive species.

**Table 3:** Summary of functional categories of genes reported to be under selection in the studies included in our database.

Genes	Organisms	Function	References
Environmental pressure	*Eurytemora affinis*, marine ascidian, sea squirts	Adaptation to salinity	Chen *et al*. (2021a); Stern & Lee (2020); Lin *et al*. (2017); Ni *et al*. (2018)
	*Oculina patagonica*, *Mytilus galloprovincialis*, round goby, European green crab, wasp spider, Colorado potato beetle, yellow fever mosquito, Asian tiger mosquito, cane toad, house crow, sea squirts, brown rat, ant, Asian house rat	Adaptation to temperature	Lin *et al*. (2017); Sherpa *et al*. (2019b); Krehenwinkel *et al*. (2015); Goubert *et al*. (2017); Leydet *et al*. (2018); Popovic & Riginos (2020); Errbii *et al*. (2021); Chen *et al*. (2021b); Yang *et al*. (2022); Kramer *et al*. (2023); Tepolt & Palumbi (2020); Konorov *et al*. (2021); Krzemińska *et al*. (2018); Selechnik *et al*. (2019); Sherpa *et al*. (2019c); Wellband *et al*. (2018); Tepolt *et al*. (2022); Chen *et al*. (2021c)
	Asian tiger mosquito, ant, Asian house rat, house crow, brown rat	Lipid metabolic regulation	Sherpa *et al*. (2019b); Goubert *et al*. (2017); Errbii *et al*. (2021); Chen *et al*. (2021b); Konorov *et al*. (2021); Krzemińska *et al*. (2018); Chen *et al*. (2021c)
	Asian house rat	Adaptation to high‐altitude hypoxia	Chen *et al*. (2021c)
Metabolism	Mealybug, tubenose goby, sweet vernal grass, common ragweed	Detoxification	Ma *et al*. (2020); Bieker *et al*. (2022); Wellband *et al*. (2018); Gould & Geber (2016)
	Pacific oyster, *Eurytemora affinis*, fall webworm, mealybug, sea lamprey	Energy metabolism	Wu *et al*. (2019); Stern & Lee (2020); Ma *et al*. (2020); Wegner *et al*. (2020); Yin *et al*. (2021)
Growth, reproduction, development	Red lionfish, common ragweed, small hive beetle	Reproduction	Burford Reiskind *et al*. (2019); Bieker *et al*. (2022); Liu *et al*. (2021)
	Common ragweed, small hive beetle, mealybug, sea lamprey, mosquitofish, guppies, marine mussel, threespine stickleback, Florida Burmese pythons	Growth and development	Ma *et al*. (2020); Rosenthal *et al*. (2021); Vera *et al*. (2016); Card *et al*. (2018); Popovic & Riginos (2020); Bieker *et al*. (2022); Liu *et al*. (2021); Yin *et al*. (2021), Yoshida *et al*. (2016); Martin *et al*. (2016)
Insecticide resistance	*Helicoverpa* moths, Colorado potato beetle, *Halyomorpha halys*, yellow fever mosquito	Insecticide resistance	Yang *et al*. (2022); Parvizi *et al*. (2023); Kramer *et al*. (2023); Sherpa *et al*. (2018); Valencia‐Montoya *et al*. (2020); Parvizi *et al*. (2023)
Immune	Common raccoon, Australian rabbits, *Eurytemora affinis*, Pacific oyster, threespine stickleback, common ragweed, marine ascidian	Immune response	Chen *et al*. (2021a); Stern & Lee (2020); Konopiński *et al*. (2023); Hodgins *et al*. (2015); Bieker *et al*. (2022); Wegner *et al*. (2020); Yoshida *et al*. (2016); Martin *et al*. (2016); Schwensow *et al*. (2017)
Others	Fall webworms, *Drosophila suzukii*, ants, *Halyomorpha halys*	Olfactory and gustatory functions	Koch *et al*. (2020); Wu *et al*. (2019); Errbii *et al*. (2021); Parvizi *et al*. (2023)
	Dry rot fungus	DNA replication and protein modification	Skrede *et al*. (2021)
	Common ragweed, marine ascidian, round goby, marine mussels, red turpentine beetle, glossy buckthorn, *Mikania micrantha*, *Helianthus annus*, *Ambrosia trifida*, *Ambrosia artemisiifolia*, *Centaurea diffusa*, *Centaurea solstitialis*, *Cirsium arvense*	Response to abiotic stressors	Chen *et al*. (2021a); Hodgins *et al*. (2015); Popovic & Riginos (2020); Bieker *et al*. (2022); Liu *et al*. (2022); De Kort *et al*. (2016); Yang *et al*. (2017); Wellband *et al*. (2018)

New environmental conditions pose significant challenges for invasive species, particularly during establishment in a new range. Specific genes under environmental selection were identified in 19 articles where selection was detected (Table 3). Genetic adaptations to temperature were found in 10 of all analysed environmental‐related studies and were key in populations invading colder climates, higher altitudes and/or facing seasonal variability (e.g. overwintering). Genes associated with lipid metabolic pathways constituted a significant subset of temperature‐related genes. Lipid metabolic regulation is involved in adaptation to fluctuating temperatures, overwintering and diapause regulation (Table 3).

According to the enemy release hypothesis, pathogens left behind during species translocation should relax selection on the host genome (Colautti *et al*., 2004). However, genes associated with the immune response were found to be under selection in seven studies. Candidate genes often were linked to virus and helminth infection or were inflammatory response‐related genes (Table 3). A response of immune‐related genes to environmental stressors was detected in the marine ascidian *Molgula manhattensis* (Chen *et al*., 2021a).

Genes involved in metabolic pathways were under selection in nine articles. Most genes were linked to carbohydrate metabolism associated with nutrient processing (Table 3). Selection of genes linked to detoxification was found in several studies (Table 3).

Insecticide resistance represents a concern for pest or vector control programs (Kramer *et al*., 2023). A signature of selection in genes linked to insecticide resistance was reported in four studies (Table 3). Additionally, selection acting on olfactory and gustatory receptors was reported in several studies. Selection of genes associated with abiotic stressors was also reported in multiple studies.

## VI. CONTRIBUTIONS OF DIFFERENT GENOMIC CHANGES IN INVASIVE SPECIES

Several studies in our database mention genetic changes other than sequence variation that could potentially impact the success of invasive populations, including genome size, whole genome duplication (WGD), gene family expansions, gene regulation, or activity of transposable elements (TE).

Studies of intraspecific genome size variation in plants suggest that a smaller amount of nuclear DNA may be a factor in successful invasions in resource‐poor environments; invasive populations were characterised by a smaller genome in European common reed grass in North America (see Fig. 2 in Pyšek *et al*., 2018), and a smaller genome size was also an important explanatory factor for differences in functional plant traits for successful invasions (see Fig. 3 in Pyšek *et al*., 2018). By contrast, allopolyploids that have evolved as a result of merging the subgenomes of different species offer evolutionary novelty through biased homoeologous gene expression and TE repression possibly enhancing adaptiveness and invasiveness associated with the species' traits and ecology (Giraud *et al*., 2021). WGD events are also a common cause of the development of increased stress resistance in plants due to advantageous changes in gene family sizes, and have contributed to invasiveness in notorious weeds (Qian *et al*., 2022). Paleoploidic history in combination with lineage‐specific and recent segmental genomic duplications can lead to rapid adaptation to new habitats, especially when gene families related to fast growth are significantly expanded (Liu *et al*., 2020b). Another key adaptation is the capacity of invasive species to establish large clonal populations, as has been shown in parthenogenically reproducing species (Gutekunst *et al*., 2018). Their evolutionary success lies primarily in the potential to use increased heterozygosity associated with polyploidy and clonal expansion for rapidly acquired adaptive advantages (Gutekunst *et al*., 2018).

Some studies used experimental approaches to demonstrate the importance of particular invasive traits, such as the upregulation of key genes, and provided additional evidence for mechanisms facilitating invasion (Wu *et al*., 2019; Rispe *et al*., 2020; Liu *et al*., 2020a; Liu *et al*., 2018; Zhang *et al*., 2020; Giraud *et al*., 2021). Studies comparing gene expression patterns between native and invasive populations under stress showed that expression responses to stress changes, indicating genetic differentiation of invasive populations, involves the rewiring of regulatory networks (Marín *et al*., 2021; Oh *et al*., 2022).

TEs, in general, generate a cost in the host genome and are usually removed or inactivated. Founder events can create periods during which the strength of purifying selection is weaker, facilitating activation and expansion of such elements, as has been demonstrated in a fungal pathogen (Oggenfuss *et al*., 2021). This can shape and diversify the genomes of invasive species. Only two studies in our database analysed TEs in the context of invasion, providing evidence for the association of some active TE families with invasive populations (Mérel *et al*., 2021; Lee & Wang, 2018). Still, the impact of TEs on fitness and adaptation or maladaptation in invasive populations remains to be studied further.

Genetic factors contributing to invasion success extend beyond SNPs and often require well‐annotated genomes and experimental testing. However, they offer a broader perspective on mechanisms of adaptation and in particular on adaptation of invasive species to new environments.

## VII. NON‐GENETIC FACTORS CONTRIBUTING TO INVASION

Our search terms were related to mechanisms that could potentially affect the genetic diversity and performance of invasive species. The collected articles also were screened for information on non‐genetic mechanisms that could explain the invasion success of particular species. Only 13 of all analysed articles mentioned non‐genetic factors as contributing to successful invasion. By qualifying the mechanism as non‐genetic, we excluded information that the invasion was human‐mediated, as invasions are inherently related to human activity. We also excluded factors that may have a genetic background, but where their elucidation was not the purpose of the study (i.e. factors that were mentioned in the text but not directly studied such as the breath of thermal tolerance or extreme insecticide resistance).

The most frequently mentioned non‐genetic factor was habitat niche congruence between native and invasive ranges (Green *et al*., 2023; Du *et al*., 2021; Sherpa *et al*., 2019c; Gutekunst *et al*., 2018), although only one of these studies performed niche comparison analysis (Sherpa *et al*., 2019c). Niche comparisons of Asian tiger mosquito invasion in Europe supported niche conservatism, suggesting that the invasive range expansion did not necessitate new genomic adaptations, although genomic adaptations were detected in invasive populations. Other mechanisms included “enemy release” in the invasive range (Bieker *et al*., 2022) and the composition of the gut microbiome (Liu *et al*., 2018).

The infrequent references to non‐genetic factors promoting invasions suggest that authors often concentrate on individual factors, and that multidisciplinary studies are rarely undertaken. Although this approach is understandable, given the time and cost demands of comprehensive studies, it complicates an holistic understanding of the invasion process. Conversely, it may suggest that mechanisms related to genomic diversity may predominate.

## VIII. DISCUSSION AND FUTURE DIRECTIONS

There is an inherent bias related to studying successful species invasions that affects our understanding of invasion mechanisms. Although it is difficult to study failed invasions, research into the causes of failed biocontrol and studies of historical data (Marsico *et al*., 2010) can provide a context in which to assess successful invasions. Such data sets are limited, but the awareness of the costs of alien species introduction is growing. According to the EU Invasive Alien Species Regulation (1143/2014), strict monitoring of all stages of species invasions must be undertaken and there is a requirement for science‐based decision making. These regulations should encourage scientists to study early stages of invasions and identify causes of varying invasion success. Below we describe the general picture emerging from the latest invasion genomics studies, present a critical assessment of gaps in the field and propose directions for future research.

The frequent role of admixture in elevating the levels of genetic diversity in novel invasive habitats is the most important finding of our study. Although admixture can be detected using data from well‐adopted and affordable techniques, such as RRS and standard population genetics tests, we argue that there should be scope for other approaches to answer more detailed questions and understand the consequences of admixture. Precise identification of the time and extent of admixture between divergent populations in the invasive range, categorisation of invasive populations according to the degree of admixture, and identification of “admixture hotspots” should be a focus in invasive species management. Moreover, studies measuring fitness change or an increase/decrease in invasiveness after admixture will be necessary to assess its role in adaptation of invasive species. We propose the term “Invasive Evolutionarily Significant Unit” (IESU) to be used in management of invasive populations. This term extends the longstanding concept of Evolutionarily Significant Unit (ESU) in conservation genetics. An ESU represents a population or a group of populations evolving independently and managed for effective species conservation (Hoelzel, 2023). We recommend designating invasive population groups or spatial areas to IESUs where a high level of admixture has possibly increased the evolutionary potential of an invasive species. RRS approaches should be adopted as an inexpensive and widely accessible tool for the identification of admixture hotspots to which proactive management actions should be directed.

We also see the need to collect and analyse time‐point series data which are crucial for detecting selection and demographic events at different stages of invasion. Time‐series sampling would allow precise detection and estimation of the frequency and timing of admixture events and bottlenecks occurring upon introduction in the new range and the tempo of evolutionary change. Additionally, they could help determine if selection occurred immediately after introduction, whether it required a lag phase, or took place during population expansion. Although we identified collected numerous studies that compared the genetic diversity of native and invasive populations, most of these were point measurements that do not allow us to evaluate precisely when a change in genomic diversity occurred. Collecting time‐series data for invasive species will be challenging as many invasions are only identified at later stages. Nevertheless, increasing awareness of the risk potentially posed by alien species, especially from rapidly spreading and fast‐reproducing groups, means that genetic monitoring of such taxa could enable the collection of these vital data.

Our review found the most common evidence of adaptation was the detection of outlier loci between native and invasive ranges. Outlier loci can be identified with a variety of genomic methods, with different selection criteria, and with or without taking into account the underlying demographic history. Such methods do not inform us about the extent of a species' response to selection but rather identify potential genetic targets of selection. In the absence of information on environmental features responsible for the change, or experimental manipulation of outlier loci, this approach does not allow biological interpretation of the selection process. Analysing selection in both invasive and native ranges, for example using GEA analysis, with respect to habitat heterogeneity, instead provides a comprehensive understanding of the role of the adaptive process in invasions. Furthermore, measurements of the level of genetic diversity that is needed for a species to adapt to environmental change can be of use when modelling species responses to climate change. Data on adaptive potential expressed as variation in functional loci and the levels of genomic diversity should be incorporated into species distribution and ecological niche modelling. Studies of native and invasive populations inhabiting differentiated habitats should provide evidence for the extent of parallel evolution in invasive populations. Invasive species could serve as excellent models for measuring and validating genetic offset, that is a measure of the mismatch in the genotype–climate association between current and future climates (Fitzpatrick & Keller, 2015), and for evolutionary rescue in the face of climate change. Invasive populations represent natural experiments, and hence enable comparisons of population fitness in new environments with fitness expected from GEA predictions.

Incorporating fitness, population performance or phenotypic data into the study of adaptations in invasive species requires controlling for environmental variation, often by using experimental designs. Identification of the most common basis of phenotypic variation encoded by multiple small‐effect loci can be achieved by controlling for environmental variation and using the genome wide association studies (GWAS) approach. GWAS allows for identification of species characteristics that are crucial for effective invasion such as dispersal, reproduction and resistance to control measures (Blackburn *et al*., 2024). We did not find evidence of *de novo* mutations being a frequent source of diversity and adaptation in invasive populations, despite previous assertions, not only for invasive species but for the process of adaptation in general (Capblancq *et al*., 2020). In our database, standing genetic variation appears to be the most commonly detected, but also most commonly investigated, source of adaptation in invasive populations. Our lack of detection of a role for *de novo* mutations could be the result of the still limited number of studies using high‐coverage WGS data.

Although the cost of WGS technologies, especially long‐read sequencing, is still prohibitive for many researchers, the number of reference genomes available is gradually increasing. Projects such as Earth BioGenome Project (EBP) or European Reference Genome Atlas (ERGA) are producing high‐quality genomic sequences across eukaryotic diversity (Formenti *et al*., 2022). Numerous invasive species still lack reference genomes (Matheson & McGaughran, 2022), but we expect a rapidly increasing availability of WGS data. Importantly, even when WGS data were used in invasion studies, the full potential of the data often was not used to elucidate the mechanisms of invasions. Fine resolution of genetic diversity and population structure provided by WGS ensures accurate estimation of effective population size (Ne), recombination rate, linkage disequilibrium or mutation load. Nevertheless, many studies using sequenced genomes relied only on SNP data. WGS data allow us to analyse structural variation (SV) that should be considered when determining local adaptation. Large‐effect genetic changes may be achieved by groups of mutations in tight genetic linkage (Yeaman & Whitlock, 2011), including mutations captured by chromosomal inversions. There is growing evidence that inversions can drive range expansions (Kirkpatrick & Barrett, 2015). Comparisons of SV among invasive and non‐invasive species could provide insights into the role of specific genome features in adaptation during invasion.

We also noted a lack of comparative genomic studies that attempted to identify genome characteristics other than SNPs underlying species invasiveness. In their new range, invasive species typically experience a new climate, availability of resources and biotic interactions. Adaptation to these new environmental conditions sometimes requires novel genetic variation that may provide new functions important for facing new environmental challenges. Such sudden shifts in selective pressures may result in large‐effect mutations (Orr, 2005). The sources of novelty are often gene duplications, resulting in the expansion of gene families and subsequent neofunctionalisation of specific genes (Salojärvi, 2019). Comparison of gene family sizes between genomes of related native and invasive species and selection acting on these genes can provide insights into how genomic novelty enhances adaptive responses (Pyšek *et al*., 2018).

Genome‐wide studies allow identification of genes and molecular mechanisms crucial for understanding the success of invasive species and for developing countermeasures (Heuertz *et al*., 2023). The application of genomic data to prevent or manage invasions has been restricted to certain groups of invasive organisms, such as in the biosurveillance of forest insect pests (Roe *et al*., 2019) or crop pests (Taylor *et al*., 2012; Wani *et al*., 2022). Examples of applications in other groups of invasive species are still limited (but see Ferreira‐Martins *et al*., 2021; Harvey‐Samuel, Ant & Alphey, 2017) and are currently under development. This application gap highlights the potential future directions of WGS, which should prioritise controlling invasive species to promote biodiversity conservation and safeguard ecosystem services.

## IX. CONCLUSIONS

(1) This systematic review of articles using genomic methods to reveal mechanisms of species invasions shows that the “genetic paradox” of invasive species is not a common phenomenon.
(2) Demographic processes, such as genetic drift and bottlenecks rarely cause a significant decrease in genomic diversity. Any reduction in genomic diversity usually was described as relatively mild and almost always resolved *via* gene flow between different invasive populations.
(3) Even where a decrease in genomic diversity between native and invasive ranges was noted, the overall level of genomic diversity in invasive populations usually remained high. Selection processes could be detected in more than half of the analysed studies, further demonstrating that reductions in diversity do not prevent adaptations to novel habitats.
(4) Despite a large number of studies involving genomics in invasion studies, there remains a need for wider application of time‐point series genomic data, the incorporation of habitat, climate and population fitness components into genomic analysis, and more frequent application of WGS data both for revealing invasion mechanisms and for invasion management.

## Supporting information


**Appendix S1.** Detailed methods.

**Database S1.** Searchable database containing query results.
