# Supplementary material for: Genomic investigations of successful invasions: the picture emerging from recent studies
Source: Biol Rev Camb Philos Soc. 2025 Feb 16;100(3):1396–418. doi: 10.1111/brv.70005 (PMC12120398; doi:10.1111/brv.70005)
Supplement: Supplementary file 1 — Appendix S1. Detailed methods. [file BRV-100-1396-s002.docx]

**Appendix S1. Detailed methods**

Our systematic review followed methodology according to the Collaboration for Environmental Evidence guidelines <https://environmentalevidence.org/standards-table/>. Published studies were identified by searching the *Web of Science* (WOS) v.5.22.1 (<https://www.webofscience.com/wos/>) database (years published: 2015–2023). We restricted our search to peer-reviewed articles and high-quality data; therefore, we searched only the WOS database and avoided grey literature. We aimed to identify studies that applied genomic methods to study invasive alien species, i.e. alien species that spread successfully after transferring into a new range and pose a threat to local ecosystems. According to the IUCN Environmental Impact Classification for Alien Taxa (EICAT), <https://iucn.org/resources/conservation-tool/environmental-impact-classification-alien-taxa> “Invasive alien species are one of the main drivers of biodiversity loss and species extinctions, and one of the most serious and rapidly growing threats to food, health and livelihood security”. Therefore, each of our searches included the terms *invasive AND genomic* combined with specific key words (identified after each question below in bold italics) to collect studies that allowed us to gather information for answering each of the questions we posed:

1. Is there is a predominant molecular footprint of adaptation in populations of species that experienced successful invasions and are specific selection events in the native species range linked to adaptation in the invasive range? ***invasive AND genomic AND (select* OR adapt* OR evol*)***
2. What is the role of demographic processes such as: admixture of divergent populations and inter-species hybridisation in creating levels of genomic diversity that allow successful invasion? ***invasive AND genomic (AND hybridization OR admixture)* and *invasive AND genomic AND (standing genetic variation OR SGV)***
3. To what extent do demographic processes that potentially decrease the genetic diversity of invasive populations, such as genetic drift and bottleneck, affect the diversity of invasive populations? ***invasive AND genomic AND (drift OR bottleneck)***
4. To what extent is genetic load detected in invasive populations? ***invasive AND genomic AND (genom* load OR genetic load OR mutation* load)***
5. How often is the genetic source of an invasion identified that enables the accurate characterisation of evolutionary processes impacting non-native populations? ***invasive AND genomic AND (invasion route* OR invasion origin*)**
6. What is the frequency of reporting specific genome characteristics connected to successful invasion? ***invasive AND genomic AND (architecture OR duplicat* OR expansion OR contraction OR rearrang*)***

The search was conducted between February 14th and 20th 2023. The total number of articles obtained during this first step was 1032. Duplicate articles were removed. The relevance of a study was first assessed by reading the title and abstract, conducted by two people independently (A.B. and J.K.). At this step, we excluded review and experimental articles and studies that did not use genomic methods. Here, ‘genomic’ was defined as whole genome data and all types of reduced representation data that employed at least several hundred markers, including mitogenomes. After this step, the number of articles remaining was 569.

Next, we read the full text of the retained articles in the database. Full-content inspection was conducted by all co-authors with studies assigned according to their professional field of interest. After this stage, our database consisted of 120 papers classified into 101 ‘population studies’ (i.e. analysing genetic diversity of invasive and/or native populations; among those 120 articles three studied only native populations of species reported as invasive in different ranges in the context of presence pre-introduction selection), 19 genome, transcriptome, or genome and transcriptome analyses (analysing single/several genomes or transcriptomes of invasive species in the context of invasion capability). Twenty-four population studies also included genome/transcriptome analysis.

While reviewing the whole text, information was collected according to the criteria specified below and summarised in the separate columns of Database S1. We aimed to create a spreadsheet addressing our research questions but also to provide future researchers with a comprehensive source of information. Due to the diverse subjects, approaches and methods applied in the selected articles, we relied on whole-text inspection and the readers’ decisions regarding the information extracted from the text.

Fragments of text, extracted directly from the articles and included in the ‘comments’ columns of Database S1 serve as an explanation or justification for the information given in the respective columns.

Below we present the screening rules in detail, with column references indicating he respective columns in Database S1.

**(1)** **Assessing pre-introduction selection events** (columns G–L) – identifying if a selection event occurred before the invasion (column G: YES/NO); if detected (YES in column G), information was recorded on general method type (column H, e.g. outlier loci, genotype–environment association (GEA)); result (column I – selection type: balancing, directional, diversifying, purifying or ‘allele frequency change’ where the type of selection was not assessed); regions, genes or groups of genes under selection (columns J and K), where possible we also added specific comments giving a wider perspective of the process detected and enabling rapid identification of specific text fragments in the articles (column L). All fragments taken directly from the article text are given in *italics*.

**(2)** **Assessing post-introduction selection events** (columns M–R) using the same criteria outlined under point 1.

**(3)** **Assessing how often genetic drift was found in invasive populations (random change in allele frequencies as a result of population establishment in a new range;** columns S and T) – identifying if genetic drift was measured or any information on the presence of genetic drift was given (column S – NO measure = no information on genetic drift was given; YES = signs of drift were detected/information on the presence of drift was given; NO = no drift detected/information on the absence of drift was given; joint shaping = both drift and selection shaped the diversity of invasive population). As a confirmation of drift, we used information on characteristic patterns of genetic diversity and/or linkage disequilibrium (LD) such as low frequency of rare alleles, increased allele frequency variance, reduction of allelic richness, increase of additive genetic variation, or direct calculation of Ne (effective population size), that arose after species translocation into invasive range. Where possible specific comments were added in column T.

**(4)** **Assessing if invasive populations experienced bottleneck (a decrease in population census as a result of population establishment in a new range;** columns U and V) identifying if the bottleneck was measured or any information on the presence of bottleneck was given (column U: NO measure = no information on bottleneck given; YES = signs of bottleneck detected/information on the presence of bottleneck given; YES (from the previous study) = authors report that bottleneck was recorded in a previous study of the same populations; NO = no bottleneck detected/information on the absence of bottleneck known from other sources and quoted by the authors; YES/NO = bottleneck was recorded in some, but not all populations); where possible, specific comments are added in column V. We used separate columns for genetic drift and bottleneck because in some of the collected studies, authors referred to one process, but not to another.

**(5)** **Assessing if invasive populations experienced genetic load** (columns W and X): column W – YES = genetic load detected; NO = no information on genetic load given; where possible, specific comments are added in column X.

**(6)** **Assessing if the study identifies the source of the invasive populations’ genetic diversity (derived from standing genetic variation *or de novo* mutations)** (columns Y and Z): we recorded any type of information where authors mention the possibility of the presence of *de novo* mutation. Specific tests detecting *de novo* mutations were often absent in our data set (column Y: NO = no information available; YES = specific information on the type of variation was given as standing genetic variation (SGV) or *de novo*; YES (only suggestion) = the possibility of *de novo* mutation was suggested by the authors), where possible, specific comments were added in column Z.

**(7)** **Information regarding a change in genetic diversity in comparison with the native population/s as given by the articles’ authors** (columns AA and AB). Column AA: increase, decrease, moderate decrease (cases where the article authors described the reduction as: “little”, “minor”, “slight” and similar terms), no change or no information; in the comments (column AB) we provide fragments of text explaining how the comparison was made (tested in the study or only general information given by the authors). For comparisons, we grouped studies into two categories: (a) where **increase, no change or moderate decrease** were noted by the article authors, these studies were treated as examples of no severe impact of translocation into a new range on the level of genetic diversity; and (b) studies that noted a **decrease** in diversity, i.e. examples of obvious reduction (cases where the authors described the reduction as: “strong”, “severe”, “high” “pronounced” and similar terms) of genetic diversity. As a number of studies were missing information on these change, we additionally collected information stated by the article authors on the level of genetic diversity of invasive populations (column AC: low, high, moderate, or no information). This information was then grouped into two categories: (a) **high and moderate** and (b) **low.** Although the level of genetic diversity of invasive populations was in some cases assessed arbitrarily by the articles’ authors, we extracted these data as an additional source of information.

**(8)** **Assessing if the source of invasion was studied** (column AD: YES/NO).

**(9)** **Assessing if the study reports multiple sources of invasion** (columns AE–AJ): columns AE, AF – YES, NO, YES from the previous study; if YES then entries in subsequent columns for methods used to detect the putative **admixture between invasive populations** (column AG), the outcome of the test (column AH: presence/absence of admixture, recurrent invasions, the presence of bridgehead populations or annotation that admixture has no impact); and the effect of admixture (column AI: increase in genetic diversity as a result of admixture, no effect; specific comments are added in column AJ where possible.

**(10)** **Assessing if hybridisation and introgression of different species genomes was recorded** (columns AK–AN), if YES (in AK) – what methods were used to detect hybridisation and introgression between invasive and other species (column AL), the outcome of the test (column AM), and additional information in the comments section (column AN).

**(11)** Quantifying how often **specific genomic characteristics** were investigated in the context of invasion (columns AO–AQ); if detected (column AO), we recorded the types of genomic characteristics (column AP) such as copy number variation, gene family expansion and contraction, genome duplication, gene expression changes, increased TE content and whole genome duplication; comments are given in column AQ.

**(12)** Quantifying how often **non-genetic factors** are reported as involved in invasion success (column AR); comments were given in column AS.

**(13)** General comments providing additional information on the study (column AT).

**(14)** Study system (columns AU and AV): column AU – e.g. invasive populations, native populations, native/invasive populations, invasive species, genome study, genome/transcriptome study); column AV – study system - grouping (population study = where multiple native and/or invasive populations were studied; genome/transcriptome study = studies of specific genomic/transcriptomic characteristics of invasive species).

**(15)** General information (columns AW–AY): studied species (column AW), type of habitat (column AX) and world region (column AY) colonised by studied populations.

**(16)** Type of genomic markers or sequencing method (column AZ).

**(17)** Taxonomy (columns BB–BQ).
